# Response to: ‘Methodological challenges for conducting case control studies to investigate the association between onchocerciasis and epilepsy including nodding syndrome’

**DOI:** 10.1093/braincomms/fcad339

**Published:** 2023-12-20

**Authors:** Arthur W D Edridge, Gasim Abd-Elfarag, Hans Verhoef, Lia van der Hoek, Michael Boele van Hensbroek

**Affiliations:** Amsterdam Centre for Global Child Health, Emma’s Paediatric Hospital, Amsterdam UMC, location University of Amsterdam, 1105 AZ Amsterdam, The Netherlands; Department of Medical Microbiology and Infection Prevention, Amsterdam UMC, location University of Amsterdam, 1105 AZ Amsterdam, The Netherlands; Amsterdam Centre for Global Child Health, Emma’s Paediatric Hospital, Amsterdam UMC, location University of Amsterdam, 1105 AZ Amsterdam, The Netherlands; Department of Neurology & Psychiatry, College of Medicine, University of Juba, P.O. Box 82, Juba, South Sudan; Division of Human Nutrition and Health, Wageningen University, 6701 AR Wageningen, The Netherlands; Department of Medical Microbiology and Infection Prevention, Amsterdam UMC, location University of Amsterdam, 1105 AZ Amsterdam, The Netherlands; Amsterdam Centre for Global Child Health, Emma’s Paediatric Hospital, Amsterdam UMC, location University of Amsterdam, 1105 AZ Amsterdam, The Netherlands

We thank Colebunders *et al*. for the critical analysis of our manuscript.^1,2^ In essence, they argue that nodding syndrome—being part of a larger entity called onchocerciasis-associated epilepsy (OAE)—is caused by *Onchocerca volvulus* and that our study failed to find an association because of methodological issues. Here, we would like to counter their arguments and provide an alternative viewpoint on the aetiology of nodding syndrome.

Many of the arguments used by Colebunders *et al*.^2^ to support a causal role for *O. volvulus* in nodding syndrome refer to studies on OAE, yet nodding syndrome is not the same as OAE, and thereby, these arguments may not be relevant. While nodding syndrome has a very specific case definition, OAE is defined as ‘a type of epilepsy that appears in onchocerciasis-endemic regions with high ongoing transmission in previously healthy children between the ages of 3 and 18 years without an obvious cause’.^3^ This definition is much broader than nodding syndrome and includes a wide variety of other epileptic disorders. Moreover, an important limitation is the exclusion of an obvious cause of epilepsy, which remains unknown in >60% of sub-Saharan African children,^4^ especially in areas with poor access to healthcare where virtually all epilepsies remain undiagnosed. This implies that in onchocerciasis-endemic regions, which apply to 30 African countries,^5^ any child with undiagnosed epilepsy may be classified as OAE.

We disagree that our study was methodologically unable to find an association with *O. volvulus*. First, Colebunders *et al*.^2^ suggest that community-directed treatment with ivermectin may have hampered our ability to detect present *O. volvulus* infection. Yet, as demonstrated by our study and one by Colebunders,^1,6^ only a minority of the study population had received ivermectin, and this proportion was similar between affected and non-affected households. Also, while community members may believe ivermectin decreases seizure frequency, this is unlikely to differ for cases and matched household controls, as all family members are usually treated at the same time. Second, it is unlikely that our association between nodding syndrome and vitamin A resulted from multivitamin supplementation. While wasting was associated with lower vitamin B_12_ concentrations, it was not associated with vitamin A. Moreover, if multivitamins were given, this would likely also increase concentrations of folate, vitamin B_12_ and vitamin B_6_, which we did not observe. Third, they argue a significant association with *O. volvulus* by serology would be found if more samples were analysed, yet we oppose extrapolating results beyond available sample sizes. Last, we disagree that the age of the nodding syndrome cases in our manuscript (median 15 years) is beyond the age range at which symptoms normally occur. The disease dynamics of nodding syndrome remain largely unknown, and while some recall bias may be present, it is unlikely that the duration of disease was mistakenly remembered to be 7 years (required to comply with the suggested age) instead of <1 year. Additionally, none of the cases in our study had a severe disease stage^7^ (all 1–3 out of 5) nor were stunted, both of which would require multiple years of disease to develop.

Based on the available evidence (reasons listed in Table 1), we consider it unlikely that *O. volvulus* is a cause of nodding syndrome. If so, the question remains why previous studies did find an association.^12^ One possibility is that *O. volvulus* is a confounder. Onchocerciasis is endemic in resource-poor settings where many other potential causal (co-)factors such as parasitic infections, malnutrition, poverty and poor access to clean water and healthcare are prevalent. Another possibility is that *O. volvulus*, or filarial infection in general, is one of the multiple component causes of nodding syndrome. For example, as filariae are known to modulate nutrient concentrations of the host,^13^ perhaps severe filarial infection combined with a specific diet, another parasitic infection, a genetic trait or another factor results in nodding syndrome. Last, *O. volvulus* infection may occur after the onset of nodding syndrome. Our study is the first to study early-onset (symptoms <1 year) cases, which found no association with *O. volvulus*,^1^ while studies of cases with much later onset did find an association.^12^ This raises the possibility that having nodding syndrome increases the risk of *O. volvulus* infection or that *O. volvulus* is associated with progression of disease.^8^

**Table 1:** Arguments opposing a causal role for *O. volvulus* in nodding syndrome

*Onchocerca volvulus* has a widespread global distribution while nodding syndrome is only present in a few hotspots.^8,9^ If a necessary cause, every individual must have been exposed to *O.* volvulus, yet not all, or only a minority of cases, are exposed.^1,8,10^ Current or recent *O. volvulus* infection was not associated with incident nodding syndrome.^1^ Animal studies of *O. volvulus* are unable to reproduce nodding syndrome–like symptoms.^11^ There is no evidence of a disease mechanism (e.g. invasion of the CSF or through cross-reactive autoimmunity) in which *O. volvulus* causes nodding syndrome.^1,9,12^

While Colebunders *et al*.^2^ refer to the World Health Organization (WHO) statement as a solid foundation for justifying public health interventions for OAE, we urge some caution, as the document specifies that ‘there currently is no formal recommendation from WHO for programmes on the topic of onchocerciasis-associated epilepsy’, and ‘a thorough analysis of the data and development of programmatic recommendations were beyond the scope of this meeting’.^14^

In the end, insufficient evidence is currently available to make any strong conclusions regarding the cause of nodding syndrome. While further aetiological case–control studies are needed (e.g. to explore novel causal hypotheses as suggested in the scientific commentary by Spencer^15^), it is also time to perform prospective intervention studies to prevent the onset or slow down disease progression in high-risk and severely affected areas. While these longitudinal studies should include *O. volvulus*, they should also consider other factors such as nutritional deficiencies and other parasitic infections. We remain with our standpoint that such interventions should only be done in research context, as otherwise their effects cannot be proven.

## Competing interests

The authors report no competing interests.

## Data Availability

Data sharing is not applicable to this article as no new data were created or analysed in this study.

## References

[1] Edridge AWD, Abd-Elfarag G, Deijs M, et al Parasitic, bacterial, viral, immune-mediated, metabolic and nutritional factors associated with nodding syndrome. Brain Commun. 2023;5(5):fcad223. 37731906 10.1093/braincomms/fcad223 PMC10507744

[2] Colebunders R, Hadermann A, Fodje J. Methodological challenges for conducting case control studies to investigate the association between onchocerciasis and epilepsy including nodding syndrome. Brain Commun. 10.1093/braincomms/fcad338 PMC10755343 38162901

[3] Hadermann A, Amaral LJ, Van Cutsem G, Siewe Fodjo JN, Colebunders R. Onchocerciasis-associated epilepsy: An update and future perspectives. Trends Parasitol. 2023;39(2):126–138. 36528471 10.1016/j.pt.2022.11.010

[4] Esterhuizen AI, Carvill GL, Ramesar RS, et al Clinical application of epilepsy genetics in Africa: Is now the time? Front Neurol. 2018;9:276. 29770117 10.3389/fneur.2018.00276 PMC5940732

[5] Frallonardo L, Di Gennaro F, Panico GG, et al Onchocerciasis: Current knowledge and future goals. Front Trop Dis. 2022;3:986884.

[6] Jada SR, Dusabimana A, Abd-Elfarag G, et al The prevalence of onchocerciasis-associated epilepsy in Mundri West and East Counties, South Sudan: A door-to-door survey. Pathogens. 2022;11(4):396. 35456071 10.3390/pathogens11040396 PMC9025150

[7] Idro R, Opoka RO, Aanyu HT, et al Nodding syndrome in Ugandan children—Clinical features, brain imaging and complications: A case series. BMJ Open. 2013;3(5):e002540. 10.1136/bmjopen-2012-002540 PMC3646179 23645924

[8] Foltz JL, Makumbi I, Sejvar JJ, et al An epidemiologic investigation of potential risk factors for nodding syndrome in Kitgum District, Uganda. PLoS One. 2013;8(6):e66419. 23823012 10.1371/journal.pone.0066419 PMC3688914

[9] Spencer PS, Mazumder R, Palmer VS, et al Environmental, dietary and case-control study of nodding syndrome in Uganda: A post-measles brain disorder triggered by malnutrition? J Neurol Sci. 2016;369:191–203. 27653888 10.1016/j.jns.2016.08.023

[10] Centers for Disease Control and Prevention (CDC) . Nodding syndrome—South Sudan, 2011. MMWR Morb Mortal Wkly Rep. 2012;61(3):52–54. 22278159

[11] Olum S, Scolding P, Hardy C, Obol J, Scolding NJ. Response to: ‘Nodding syndrome, many questions remain but we can prevent it by eliminating onchocerciasis’. Brain Commun. 2021;3(1):fcaa229. 33502386 10.1093/braincomms/fcaa229 PMC7811753

[12] Abd-Elfarag GOE, Edridge AWD, Spijker R, Sebit MB, van Hensbroek MB. Nodding syndrome: A scoping review. Trop Med Infect Dis. 2021;6(4):211. 34941667 10.3390/tropicalmed6040211 PMC8703395

[13] Storey DM . Filariasis: Nutritional interactions in human and animal hosts. Parasitology. 1993;107(S1):S147–S158. 8115179 10.1017/s0031182000075570

[14] Report of the sixth meeting of the WHO Onchocerciasis Technical Advisory Subgroup: virtual meeting, 19-21 December 2022. Accessed 4 October 2023. https://www.who.int/publications/i/item/9789240071469

[15] Spencer PS . New clues to the elusive aetiology of nodding syndrome. Brain Commun. 2023;5(5):fcad236. 37731902 10.1093/braincomms/fcad236 PMC10507742

